# Types of *Artemisia* pollen season depending on the weather conditions in Wrocław (Poland), 2002–2011

**DOI:** 10.1007/s10453-013-9304-4

**Published:** 2013-05-26

**Authors:** Małgorzata Malkiewicz, Kamilla Klaczak, Anetta Drzeniecka-Osiadacz, Justyna Krynicka, Krzysztof Migała

**Affiliations:** 1 Institute of Geological Sciences, University of Wrocław, Wrocław, Dolnośląskie Poland; 2 Institute of Geography and Regional Development, University of Wrocław, Wrocław, Dolnośląskie Poland

**Keywords:** Aeroallergens, Aerobiology, Crop Heat Units, Modelling of pollen seasons, Mugwort, Regression analysis

## Abstract

The aim of the study was to characterise *Artemisia* pollen season types according to weather conditions in Wrocław (south-western Poland) in the years 2002–2011. Over the period analysed, the start date of the pollen season (determined by the 95 % method) ranged from 10 July 2002 to 28 July 2010. The start date of the pollen season can be determined by using Crop Heat Units (CHUs). During the period 2002–2011, the *Artemisia* pollen season started after the cumulative value of CHUs had reached 2,000–2,100 °C. The three distinguished types of *Artemisia* pollen season are best described by the frequency of weather types defined by the type of circulation, mean daily air temperature, and the occurrence of rain. The variation in these factors affected the dynamics of the pollen season. The noteworthy frequency of days with rain and high seasonal sum of precipitation totals as well as the dominance of cyclonic weather from the westerly direction had an impact on the extension of the pollen season. The meteorological factors that directly affect pollen release and transport primarily include air humidity, expressed as vapour pressure (*r* > 0.3, *p* < 0.01), temperature(*r* from 0.2 to 0.4, *p* < 0.01). The relationships between averaged meteorological data and daily pollen concentration were stronger (*r* > 0.5, *p* < 0.01). Based on the correlation analysis, the meteorological variables were selected and regression equations were established using stepwise backward regression analysis.

## Introduction

*Artemisia* L. is considered to be a late summer plant. In north-eastern Europe, its flowering and pollen release occur from the last week of July until the last week of August (Spieksma et al. 1980; Nilsson and Palmberg-Gotthard 1982; Goldberg et al. 1988). The genus *Artemisia* L. produces ca. 689 pollen grains per anther (Subba-Reddi and Reddi 1986) which are an major cause of allergies in Central and Eastern Europe. The frequency of allergies among the European population is between 3 and 15 % (Ipsen et al. 1985; D’Amato et al. 1998).

Poland is one of the countries with the highest allergy incidence rates (Heinzerling et al. 2009). Furthermore, a higher rate of disease symptoms (twice higher) can be observed among the urban population compared to people living in rural areas. This can be associated with a high concentration of air pollutants in urbanised areas, which are mainly generated by road transport (sulphur and nitrogen oxides, dust, ozone, diesel exhaust particles—DEPs). These circumstances lead to increased respiratory sensitivity to environmental factors, including aeroallergens (Kinney et al. 2000; de la Guardia et al. 2006; Tombácz et al. 2007; Komorowski 2012).

The spread, vegetative growth, flowering and fruiting of plants are greatly affected by the geographic location and local climate. Generally, the further north, the shorter the plant growing season is, however, numerous aerobiological observations and meteorological analyses prove that weather conditions have a greater effect on the duration and intensity of pollen release than their location. Meteorological variables, such as mean air temperature, sunshine duration, total precipitation, relative humidity, wind speed and direction, are mentioned as factors that significantly influence the duration and intensity of pollen release as well as airborne pollen concentrations (Giner et al. 1999; Spieksma et al. 2003; Puc 2006, 2009; Dąbrowska and Chłopek 2007; Stach et al. 2007; Grewling et al. 2012; Melgar et al. 2012).

Attempts to create a forecasting model which would predict pollen concentrations and characterise the pattern of the mugwort pollen season have already been being undertaken for a long time (Koivikko et al. 1986; Wolf et al. 1998; Stach et al. 2007). Some of the first attempts were related to the development of a mathematical model that would include only basic meteorological data (extreme temperatures, pressure and precipitation) in averaging mainly 1-day periods (Wolf et al. 1998). The next forecasting models, also those developed for Poland, included a larger number of meteorological factors, that is wind speed and direction, air humidity, sunshine duration, structure of the atmospheric boundary layer as well as the impact of terrain conditions and long-range transport (Giner et al. 1999; Puc 2006; Laursen et al. 2007). The pollen of *Artemisia* L. is one of the three major aeroallergens in Central and Eastern Europe. The epidemiological research shows a high frequency of people suffering from allergy-induced respiratory diseases in the population of Wrocław, including persons exhibiting asthma symptoms (nearly 28 %). Thus, pollinosis is an important factor that deteriorates the quality of life of the urban population (Komorowski 2012). It is therefore necessary to create a forecast model for the growing season, which would be a forecasting tool for people with pollinosis symptoms. The following analysis can be one of the examples of such a model.

## Materials and methods

### Study area

The pollen data and meteorological variables were gathered in Wrocław during the period 2002–2011.

**Fig. 1:**
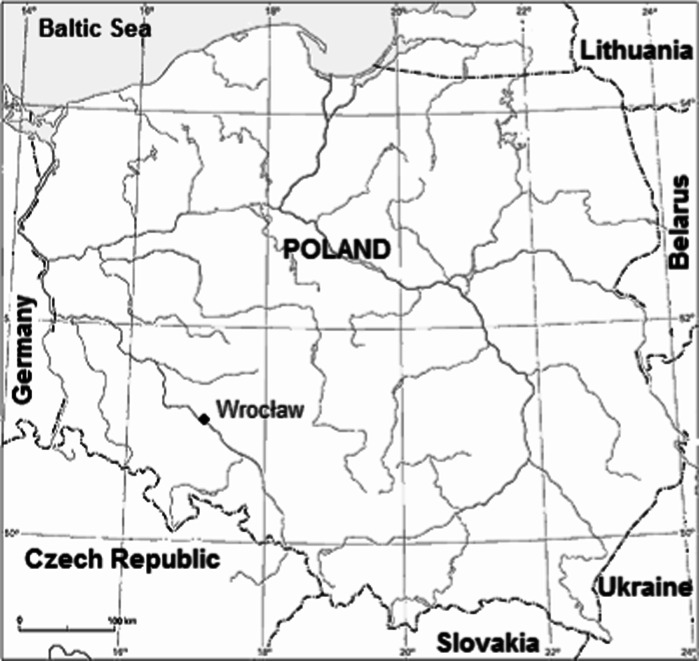
Location of the measurement stations

Wrocław (51°07′N, 17°02′E) is located in south-western Poland (Fig. 1), it occupies an area of 293 km^2^, and its population is 633,000 (CSO 2011).

Due to Wrocław’s geographic location at the foreland of the Sudety Mountains (Kondracki 2001) and the existing climatic conditions (the city is thermally privileged), the term “warm region of Wrocław and Opole” is applied to this location. Wrocław is characterised by the typical features of a transitory temperate climate zone, with predominance of oceanic influences and westerly atmospheric circulation being quite evident in this city (Dubicka 1994). The Köppen–Geiger Climate classification system places Wrocław in the oceanic climate (Cfb) (Kottek et al. 2006). The highest wind speeds are recorded from the westerly sector and the lowest ones from the northerly sector, and moreover, higher wind speeds occur during the cold season of the year. Winters are mild and short, while the springs start early. The growing season lasts 226 days (Woś 1999; Dubicki et al. 2002).

In addition to the meteorological conditions, the topographic features and the type of land cover are important factors creating the specific features of the Wrocław urban climate. Wrocław is a city with the highest urban green space rate per inhabitant (ca. 25 m^2^) in Poland. It is characterised by an extensive river network, numerous islands, green belts, parks, and allotment garden areas; they all form a unique mosaic, together with residential and industrial areas, as well as transport infrastructure.

In accordance with the Corine Land Cover (2004), various kinds of technical land use cover 42.1 % of the city area (housing estates, roads, industrial and warehousing buildings). The remaining part of city (57.9 %) consists of natural, semi-natural (urban green space), agricultural areas and water (Drzeniecka-Osiadacz et al. 2010). It may, however, be assumed that a large part of this land is not used agriculturally and is wasteland. Wasteland and heavily human-transformed areas are predominantly colonised by ruderal plants; hence, such areas are a potential source of *Artemisia* pollen emission. *Artemisia vulgaris*, *Artemisia campestris* and *Artemisia absinthium* all occur in Wrocław (Zając and Zając 2001).

### Aerobiological data

The investigations were carried out using the volumetric method based on Hirst’s construction (Hirst 1952). Daily pollen concentrations were monitored from January to October over the 2002–2011 growing seasons using a Burkard 7-day volumetric pollen trap. The sampler (borrowed from the Centre for Research on Environmental Allergens) was placed in the city centre, on the roof of the building of the Institute of Geological Sciences of the University of Wrocław, at a height of about 20 m above ground level. In the immediate vicinity of the sampling site, there is a dense urban built-up area and scanty patches of greenery. From the south, the building is surrounded by an alley of plane trees, while several horse-chestnut trees and small birches grow to the north of the building.

Airborne pollen counts were analysed following the recommendations of the International Association for Aerobiology (Mandrioli et al. 1998). Cylinder with tape was replaced at 09:00 a.m. each Thursday. Pollen grains were counted under a light microscope along 4 longitudinal transects and expressed as the number of pollen grains per m^3^ of air (p m^−3^). Daily pollen concentration represents pollen count from midnight to midnight. The 95 % method was used to determine the start and end dates of the pollen seasons (Emberlin et al. 1993).

To classify the pollen seasons, cluster analysis was employed, including the non-hierarchical method of multi-feature clustering—the k-means method using Statistica software.

To determine the correlations between daily pollen concentration p and meteorological factors, standardisation was performed using ln(*p* + 1). The assumption of normality of the pollen data was confirmed by means of the Kolmogorov–Smirnov test.

### Meteorological data

The meteorological data used in the analysis were collected at the Meteorological Observatory of the Department of Climatology and Atmospheric Protection, University of Wrocław. The Observatory is located in the eastern part of Wrocław, near the large Szczytnicki Park, about 5 km from the city centre. The basic data used in the analysis were as follows: air temperature [daily mean *T* (°C), minimum *T*_min_ (°C), maximum *T*_max_ (°C) and daily amplitude *A* (°C)], vapour pressure *e* (hPa), atmospheric pressure *P* (hPa), mean wind speed *v* (m s^−1^), sunshine duration SD (h) and daily sum of precipitation *R* (mm). In reference to WMO standards, the sampling of meteorological data was started at 00:00 UTC every day, and then the daily characteristics were calculated. In addition, the calendar of circulation types was used (Niedźwiedź 2011). These meteorological variables were selected in order to analyse any connections between mugwort pollen concentration in distinguishing seasons.

The basic agro-climatic indices, that is growing degree days (GDD), sum of active temperatures (SAT), Crop Heat Units (CHUs) and HTI (Selyaninov’s Hydrothermal Coefficient), were used to describe hydrothermal conditions affecting plant growth (Garcia-Mozo et al. 2000; Thavaprakaash et al. 2007; OMAFRA 2009). The CHU and HTI indices were considered to be the best to characterise thermal and humidity conditions prevailing in the growing period preceding the pollen season.

The CHUs are based on a similar principle to GDDs, but the way of calculation is quite different. Daily CHUs are calculated from minimum and maximum temperatures. There are separate calculations for day and night. The daytime relationship uses 10 °C as the base temperature, while the night time one uses 4.4 °C. Accumulated Crop Heat Units (CHUs_acc_) were used to determine the influence of thermal conditions throughout the whole season. The value of 1.03 was accepted as the beginning of the period for which accumulated CHUs were calculated. The minor change was introduced to general assumptions of calculating CHU. CHUs_acc_ were accumulated by adding each day’s CHUs contribution as the season progresses, but the days with mean daily temperature above 5 °C and daily maximum temperature above 10 °C were take into account even though minimum of temperature was below 4.4 °C.

The raw data were processed, so the data set contained *N*-day means and sums of meteorological observation data (where N equals: 5, 7, 10, 12, 14, 20, and 30 days) before the date of pollen concentration measurement.

The relationships between individual meteorological factors and seasonal pollen variation were evaluated using cluster analysis. The analysis was carried out for the selected meteorological parameters (temperature, precipitation and types of atmospheric circulation) and their combinations. Furthermore, Pearson’s correlation analysis was performed for each individual pollen season. The meteorological variables characterised by the highest correlation coefficient (statistically significant at *p* < 0.01) were used to create a regression model describing the variation in daily *Artemisia* pollen concentration. For this purpose, multiple stepwise regression was used.

## Results and discussion

### Characteristics of the pollen season

The present study shows that the *Artemisia* pollen seasons started in the second half of July and usually lasted until the end of August (in the case of the years 2005, 2006 and 2010 even until the end of September). The average duration of the *Artemisia* pollen season for the 10-year study period was 39 days. The maximum values of daily concentrations in individual years varied and ranged 51–223 p m^−3^. The lowest maximum values, which were recorded in 2010 (51 p m^−3^) and 2006 (58 p m^−3^), were observed during the longest *Artemisia* pollen seasons (62 and 45 days). In turn, the highest value, recorded in 2008 (223 p m^−3^), occurred during one of the shortest pollen seasons in the period under analysis. The maximum concentrations of *Artemisia* pollen occurred between day 212 and day 230 of the year. The daily maximum was recorded earliest in 2002, at the end of July. But in 2006, the peak was clearly delayed compared to the other seasons, since the maximum concentration occurred as late as 18 August. In most years of the study, the seasonal pollen index (SPI) was more than 2,000 grains. The SPI was at a much lower level, less than 1,000 grains, in only 3 years (2006, 2010, and 2011).

**Fig. 2:**
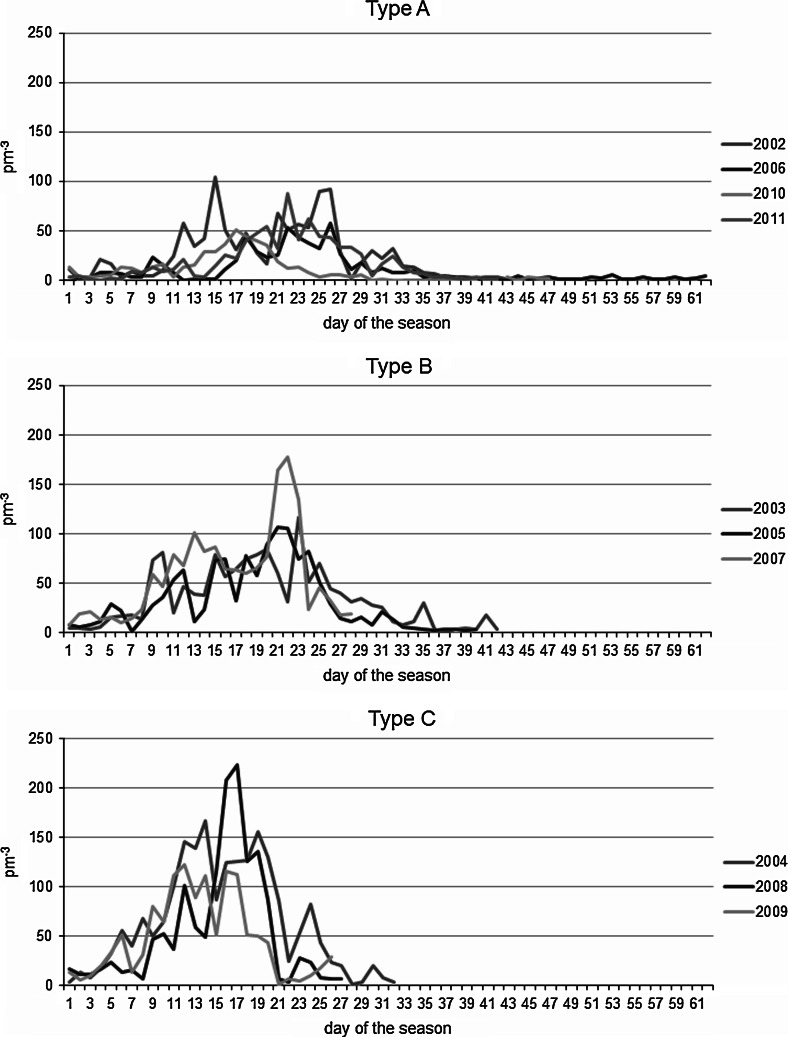
The course of pollen concentration in the distinguished pollen season types for *Artemisia* L

Due to the significant differences in the pattern of the *Artemisia* pollen seasons during the 10-year study period, an attempt was made to classify them using an objective method of multi-feature clustering—k-means. The classification of the pollen seasons was based on the number of peaks with maximum pollen concentrations, the date of maximum occurrence, calculated from the start of the season and the duration of the post-peak period. Three pollen season types were distinguished for *Artemisia* L. (Fig. 2). The largest number of pollen seasons was classified as type A (the years 2002, 2006, 2010 and 2011). Relatively low daily concentrations (50–100 p m^−3^) and long post-peak periods are characteristic for this type of pollen release. The maximum daily concentrations are spread over time and occur around the 20th day of the season. Type B comprises the years 2003, 2005 and 2007; in this type, the maximum daily concentrations occur on average around the 22nd day of the season, and they reach a value of more than 100 p m^−3^. The period of post-peak low concentrations is short. A short pre- and post-peak period is characteristic of type C (2004, 2008 and 2009); the daily concentrations of pollen grains recorded for this type are the highest, above 120 p m^−3^. They occur very early on average around the 17th day of the season.

### Characteristics of meteorological conditions

The analysis of the meteorological conditions allows one to characterise the growing seasons of *Artemisia* L. in particular years.

The years 2002–2012 were very warm; compared to the long-term period 1971–2000 (mean temperature 8.8 °C), these years were characterised by an increase in mean annual temperature to 9.5 °C. July was the warmest month in the 10-year period under analysis, while January was the coldest. The year 2007 was particularly warm, with mean annual temperature of 10.4 °C and the highest mean temperature in the spring months (11.2 °C). The years 2006 and 2010 were the coldest in the period analysed taking into account the spring temperatures (Table 1).

**Table 1:** Characteristics of meteorological conditions in 2002–2011

Period	Years	*P* (hPa)	*T* (°C)	*T*_max_ (°C)	*T*_min_ (°C)	*T*_min+5_ (°C)	*v* (ms^−1^)	*e* (hPa)	*U* (%)	SD (h)	*R* (mm)
Spring (March, April, May)	2002	1,001.3	10.6	16.6	5.9	3.0	1.6	9.3	71.0	515.7	83.7
	2003	1,004.9	9.4	15.6	4.3	1.7	1.8	8.2	67.9	606.3	112.4
	2004	1,001.5	9.3	14.6	4.7	2.5	1.9	8.1	69.4	427.3	108.6
	2005	1,001.2	8.6	14.7	3.6	1.0	1.8	8.2	70.1	574.0	154.7
	2006	999.0	8.3	13.6	3.9	0.8	1.7	7.9	71.8	458.1	93.1
	2007	1,001.3	11.2	17.6	5.4	1.3	2.3	9.2	68.9	645.6	110.5
	2008	997.4	9.6	14.9	4.2	1.2	2.0	8.2	69.6	533.9	169.7
	2009	1,002.2	10.4	16.0	4.4	1.7	1.9	8.2	66.5	610.4	141.5
	2010	1,001.8	8.8	13.9	3.9	1.9	1.9	8.3	70.4	509.6	224.2
	2011	1,006.5	10.1	16.4	3.6	0.5	1.7	8.1	63.1	677.0	100.5
Summer (June, July, August)	2002	1,000.3	19.8	26.7	14.7	12.1	1.4	16.0	71.8	664.6	224.3
	2003	1,001.3	20.1	26.9	14.5	11.9	1.6	14.5	64.3	766.6	107.9
	2004	1,000.5	18.4	25.0	13.4	10.7	1.7	14.2	69.3	613.5	135.8
	2005	1,001.1	18.1	24.2	13.1	10.7	1.6	14.5	71.6	702.3	213.9
	2006	1,001.5	19.8	26.0	14.2	11.2	1.6	15.2	68.6	756.8	259.8
	2007	998.5	19.2	25.3	14.1	9.7	1.8	15.8	72.6	702.2	227.9
	2008	1,001.4	19.0	25.4	13.1	10.5	1.8	13.7	63.2	750.1	183.8
	2009	1,002.3	18.3	24.2	12.7	10.5	1.7	14.8	71.0	686.7	320.6
	2010	1,001.3	19.5	25.4	13.4	11.1	1.4	15.0	68.0	777.7	215.6
	2011	1,001.3	18.9	24.5	13.2	11.9	1.7	14.3	65.3	648.2	285.6
2002–20011	Spring	1,001.7	9.6	15.4	4.4	1.6	1.8	8.4	68.8	555.8	129.9
	Summer	1,000.9	19.1	25.4	13.6	11.0	1.6	14.8	68.6	706.9	217.5
	Autumn	1,002.9	9.2	14.6	5.1	2.7	1.6	9.7	80.9	352.5	117.2
	Winter	1,002.7	0.2	3.5	−2.7	−4.6	2.2	5.3	81.5	160.5	121.7
	Year	1,002.1	9.5	14.7	5.1	2.7	1.8	9.5	74.9	1,775.6	586.3

*P* atmospheric pressure, *T* air temperature, *T*_max_ maximum temperature, *T*_min_ minimum temperature, *T*_min+5_ minimum temperature near the ground, *e* vapour pressure, *v* wind speed, *U* relative humidity, SD sunshine duration, *R* sum of precipitation

The growing season during the period 2002–2011 started in March, on average, whereas in 2002, the average temperature of February was already 5.0 °C and that of March 5.4 °C.

Over the last 10 years, there was also an increase in annual rainfall sum (586.3 mm) compared to the period 1971–2000 (575.5 mm). The increase in precipitation is most pronounced during winter season. On average, about 45 % of days annually experienced greater than 0.1 mm of precipitation. Over the entire year, the most common forms of precipitation were light or moderate rain. The maximum amount of rainfall was at the end of July and in August (Drzeniecka-Osiadacz et al. 2010). The highest annual sum of precipitation occurred in 2006 and 2010. On the other hand, the year 2002 was characterised by low precipitation both in the winter and in the spring season (less than 85 mm), but the maximum rainfall was in August (>120 mm).

The sunshine duration recorded was on average higher by 200 h (a total of 1,775 h) than in the long-term period (1,500 h) used for reference, and this increase was noted mainly during the summer season. For nearly 30 years, an increase in sunshine duration has been observed in Wrocław, which is associated with a reduction in cloud cover during the summer and spring period. In the period analysed, the highest values of sunshine duration were recorded for the spring in 2007 and 2011 as well as for the summers in 2003 and 2010.

Wind speed and direction determine the distribution of pollen grains released from anthers, which affect the level of concentrations during pollen seasons; hence, the analysis of anemometric conditions is of special importance for the summer months. The highest average wind speed in the summer months was observed in 2007 (1.8 ms^−1^), while the lowest one in the years 2002 (1.4 ms^−1^) and 2010 (1.4 ms^−1^). The mean values of atmospheric pressure did not show large differences in annual and seasonal variation (Table 1).

### The role of meteorological conditions during pollen seasons

*Artemisia* pollen levels in the atmosphere are a result of three processes: production, release and dispersal. Various meteorological parameters have different levels of importance during these stages. The start dates of the growing season, heat inflows and the occurrence of (excess or deficit) rainfall during plant growth and also during pollen release are particularly important here.

**Fig. 3:**
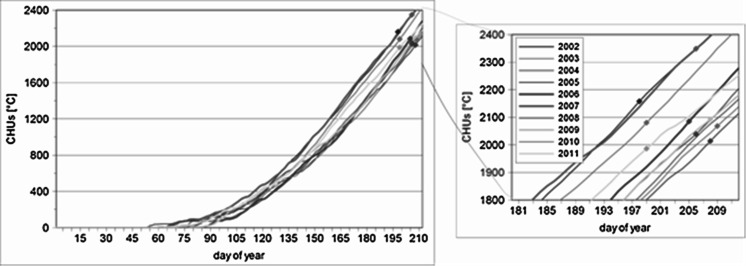
The values of CHUs_acc_ in particular years with the indicated start of the pollen season

Over the period analysed, the start date of the pollen season (determined by the 95 % method) ranged from 10 July 2002 to 28 July 2010. The pollen season start date can be best determined by using CHUs. During the period 2002–2011, the *Artemisia* pollen season started after the cumulative value of CHUs had reached 2,000–2,100 °C (Fig. 3). The year 2007, in which the pollen season began later, is the only exception here. This was caused by heavy precipitation events; 88 mm of rain was recorded in the first half of July, and this amount of rain exceeds the mean sum of precipitation for July.

According to cluster analysis, the three distinguished types of *Artemisia* pollen season are best described by the frequency of weather types defined by the type of atmospheric circulation, mean daily air temperature and the occurrence of rain. The variation in these factors affects the dynamics of the pollen season.

The significant frequency of days with rainfall and high seasonal rainfall sums as well as the dominance of cyclonic weather from the westerly direction had an impact on the extension of the pollen season in type A. On average, the highest pollen concentrations (>30 p m^−3^) for particular types of atmospheric circulation occurred on days with cyclonic circulation and an inflow of air masses from the E–SW sector.

During the pollen season of type B (intermediate type), the highest average pollen concentrations (>70 p m^−3^) were recorded on days with Na (North anticyclonic) and SWa (South–West anticyclonic) circulation as well as on days with cyclonic circulation from the E–SE sector.

In the season of type C, the high frequency of calm, warm anticyclonic weather resulted in a shorter pollen season with an unimodal characteristic pattern with a clear seasonal peak. In type C, the variation in the average pollen concentration, depending on the type of circulation and direction of air mass movement, did not show the significant differences (Table 2).

**Table 2:** Characteristics of meteorological and synoptic conditions during the pollen seasons

	Meteorological conditions during pollen seasons.	Frequency of circulation types (anticyclonic and cyclonic) during the pollen seasons^a^
Type A (2002, 2006, 2010, 2011)	High frequency (49 %) of days with rain (mean seasonal precipitation sum above 150 mm, mean HTI above 1.6), high frequency of days with a low pressure system (>50 %), low temperature—below the long-term seasonal average (except for the year 2002 which was very warm), average daily sunshine duration below 7 h, large variation in pressure during the season, a relatively low percentage (30 %) of days with a cold front compared to other frontal events.	
Type B (2003, 2005, 2007)	Moderate frequency (34 % throughout the whole season, but 42 % in July) of days with rain (mean seasonal total precipitation from 40 mm up to 90 mm), heavy rain at the end of the pollen season, mean HTI about 0.8, mean seasonal temperature and mean seasonal minimum temperature above the long-term seasonal average, average daily sunshine duration about 8 h.	
Type C (2004, 2008, 2009)	Low frequency (34 % throughout the whole season, but only 10 % in July) of days with rain (mean seasonal total precipitation from 24 mm up to 69 mm), mean HTI about 0.9, high frequency (56 %) of days with a high pressure system, low frequency (35 %) of days with frontal events (except for 2009), mean seasonal temperature above the long-term seasonal average, large daily amplitude, average daily sunshine duration about 8–10 h.	

^a^Central anticyclonic (Ca) and central cyclonic (Cc), peak of high pressure (Ka), trough of low pressure (Bc)

The correlation analysis shows varying degrees of relationship between pollen concentrations in the distinguished pollen season types and meteorological conditions. As regard the individual factors, these relationships are the strongest in type C and the weakest in types A and B (Tables 3, 4). In the case of type B, the pattern of pollen release in 2007 is important, because in this year, the pollen season started later and very rapidly, due to rainfall events just before pollen release.

**Table 3:** Coefficients of correlation between meteorological conditions and *Artemisia* pollen concentration on a given day *p* and on the next day (*p* + 1N)

Variables	Type A		Type B		Type C	
	*p*	*p* + 1d	*p*	*p* + 1d	*p*	*p* + 1d
*p* + 1d	0.89**	x	0.93**	x	0.94**	x
*p* + 5d	0.77**	x	0.85**	x	0.83**	x
*p* + 10d	0.61**	x	0.70**	x	0.65**	x
P (hPa)	−0.24**	−0.27**	−0.05	−0.04	−0.08	−0.10
R (mm)	−0.02	0.04	−0.05	−0.02	−0.05	0.01
T (°C)	0.20**	0.18**	0.31**	0.30**	0.42**	0.41**
T_max_ (°C)	0.18**	0.16**	0.28**	0.28**	0.40**	0.40**
T_min_ (°C)	0.30**	0.29**	0.25**	0.27**	0.31**	0.31**
T_min+5_ (°C)	0.29**	0.28**	0.29**	0.31**	0.32**	0.31**
v_max_ (ms^−1^)	−0.11*	−0.11**	−0.12*	−0.12*	−0.11*	−0.12**
e (hPa)	0.32**	0.32**	0.30**	0.30**	0.35**	0.36**
SD (h)	−0.06	−0.07	0.08	0.05	0.22**	0.20**

*p* + *N*_(1–10)_ d—pollen concentration *N* days later

** Statistically significant at *p* < 0.01, * statistically significant at *p* < 0.05

**Table 4:** Coefficients of correlation between meteorological conditions for different averaging periods and *Artemisia* pollen concentration

Variables	Averaging period	Type A	Type B	Type C
e (hPa)	5	0.40**	0.38**	0.46**
	7	0.44**	0.40**	0.49**
	10	0.48**	0.44**	0.51**
	12	0.49**	0.45**	0.52**
	14	0.49**	0.47**	0.53**
	20	0.41**	0.36**	0.50**
	30	0.51**	0.58**	0.49**
P (hPa)	14	−0.46**	−0.09	−0.13*
T (°C)	30	0.42**	0.50**	0.63**
T_min_ + 5 (°C)	7	0.43**	0.42**	0.47**
	10	0.49**	0.46**	0.51**
	12	0.51**	0.48**	0.54**
	14	0.53**	0.51**	0.56**
	30	0.62**	0.65**	0.68**
T_min_ (°C)	10	0.45**	0.36**	0.50**
	12	0.46**	0.37**	0.52**
	14	0.48**	0.40**	0.55**
	30	0.57**	0.53**	0.67**

** Statistically significant at *p* < 0.01, * statistically significant at *p* < 0.05

The relationships between meteorological factors and pollen concentrations are often not clear, the impact of meteorological factors is observed to be stronger in the averaging periods longer than 1 day (Table 3). These meteorological variables influence not only the pollen release process, but they also generally affect plant condition and pollen production.

The meteorological factors that directly affect pollen release and transport primarily include air humidity, expressed as vapour pressure (*r* > 0.3), sunshine duration (only in type C), temperature and wind speed (Table 4). The effect of rainfall intensity is not noticeable, since the values of the scavenging coefficients determined on a daily basis are both positive and negative. The obtained results are in agreement with the literature data (Puc 2006; Laursen et al. 2007). The highest correlation coefficients (*r* > 0.4 for temperature) between daily pollen concentration and meteorological conditions on a same day and previous day occurred for type C, which was characterised by a unimodal pollen concentration pattern. Generally, an increase in mean daily air temperature above the threshold value of ca. 20–22 °C (hot and dry periods lasting for several days) caused a decrease in pollen concentrations. Moreover, the syntactical analysis demonstrates the autocorrelation relationship of pollen concentrations, associated with the gradual maturation of anthers.

The analysis of meteorological conditions over averaging periods longer than 1 day may lead to the identification of stronger correlations between weather conditions and pollen concentration due to the long process of pollen grain formation and maturity (Wolf et al. 1998; Giner et al. 1999). Apart from the analysis of day-to-day relationships, averaged meteorological conditions were also evaluated for different time intervals. The statistical analysis showed an improvement in the strength of correlation between daily pollen concentration and average meteorological conditions for the periods of 12, 14 and 30 days (Table 4). The effects of air temperature, primarily temperature at ground level and air humidity are the most important factors here. However, a significant impact of the sunshine duration and precipitation sum was not observed.

Based on the correlation analysis, the meteorological variables were selected and regression equations were established using stepwise backward regression analysis (Table 5). The meteorological variables describe the variation in daily concentrations of *Artemisia* pollen in about 50 %. In the case of type C, the regression analysis shows that the meteorological variables (temperature, vapour pressure, sunshine duration) averaged for 12–14 days before the releasing pollen are of key importance for daily pollen concentrations. In the case of the seasons with a large number of days with rain (such as type A and B), the daily pollen concentration could be better explained by averaging a longer period than in type C (e.g. up to 30 days before pollen release) for the meteorological factors .

**Table 5:** Multiple linear regression analysis

	Regression equation	*R*_adj_^2^	*F*	*F*_sign_	STD
Type A	ln(*p*) = 0.074*e_14_ − 0.007*SD_14_ − 0.08*P_30_ +18.76*e^T_min+530_ + 73.8	0.51	126.69	1.99E−73	0.86
Type B	ln(*p*) = −0.09*v_0_ + 0.41*e_30_ + 0.31*T_min+530_ − 7.05	0.51	119.48	1.72E−52	1.08
Type C	ln(*p*) = 0.24*e_14_ + 0.16*T_min+514_ + 0.009*SD_14_ − 5.71	0.43	84.80	1.15E−40	1.14

*P* atmospheric pressure, *T*_min+5_ minimum temperature near the ground, *e* vapour pressure, *v* wind speed, SD sunshine duration; the subscripts refer to the averaging period

## Conclusions

In the period 2002–2011, differences were observed in the start and end dates of pollen release as well as in the variability of daily pollen count of the *Artemisia* within pollen seasons. These differences resulted from the meteorological conditions and the characteristics of the growing season. The average duration of the mugwort pollen season in Wrocław was 39 days. The beginning of this period was in the second half of July, while its end as late as the first days of September. The analysis performed made it possible to distinguish 3 types of *Artemisia* pollen season differing in weather characteristics and pollen release dynamics. For each type, a regression equation was also derived which included the most essential meteorological parameters and which can be used for forecasting purposes.

The analysis showed that statistically vapour pressure, air temperature and wind speed have the greatest importance for pollen concentrations recorded, in particular for longer averaging periods (a dozen or so days and monthly periods). However, precipitation amount was not shown to be significant.

An important element of the presented model is the inclusion in pollen forecasting of agro-climatic indices that thus far have not been included in other studies, namely CHUs, an indicator that performs well. The value of accumulated CHUs at a level of 2,000–2,100 °C is associated with the onset of *Artemisia* pollen release.

The present study shows the importance of the selected meteorological parameters, but also the effect of the dynamics of the growing season and of the pollen season on *Artemisia* pollen concentrations observed. Moreover, the study demonstrates the need to analyse data from not only short averaging periods (mainly daily periods), but also the means for longer periods (12 and 14 days) and monthly periods, which give higher correlation coefficients. Due to insufficiency of pollen data, the regression models were not tested, so it highlights the area of future studies.
